# Investigation of sociodemographic, temporal, and meteorological heterogeneity in the short-term blood pressure response to air pollutants

**DOI:** 10.1186/s12940-026-01275-x

**Published:** 2026-02-28

**Authors:** Dikshya Maharjan, Sanjeev Bista, Anaïs Teyton, Rosa Maria Bruno, Andrea Montanari, Dan Zou, Giovanna Fancello, Tarik Benmarhnia, Basile Chaix

**Affiliations:** 1 Faculté de Médecine Saint-Antoine, Sorbonne Université, INSERM, Institut Pierre Louis d’Epidémiologie Et de Santé Publique (IPLESP), NEMESIS Team, 27 Rue Chaligny, Paris, 75012 France; 2 Department of Cancer Epidemiology and Prevention Research, Alberta Health Services, Calgary, AB Canada; 3 Department of Oncology, Cumming School of Medicine, University of Calgary, Calgary, AB Canada; 4 Herbert Wertheim School of Public Health and Human Longevity Science, University of California, San Diego, La Jolla USA; 5 School of Public Health, San Diego State University, San Diego, USA; 6 Scripps Institution of Oceanography, University of California, San Diego, La Jolla USA; 7 INSERM U970 Team 7, Paris Cardiovascular Research Centre–PARCC, Université de Paris, Paris, France; 8 AP-HP, Pharmacology Unit, DMU CARTE, Hôpital Européen Georges Pompidou, Paris, France; 9 Department of Geography, Barefield College of Arts and Sciences, The University of Alabama, Tuscaloosa, AL 35487 USA; 10 Irset Institut de Recherche en Santé, Environnement Et Travail, UMR-S 1085, Inserm, University of Rennes, EHESP, Rennes, France

## Introduction

Air pollution causes roughly 7 million premature deaths every year globally [31]. According to World Health Organisation, 99% of the global population reside in areas with air pollution levels exceeding the guidelines [33]. With accelerating climate change, air pollution, especially ozone concentrations, and its adverse consequences on health are being amplified, particularly in urban areas like Paris [2].

Long-term effects of air pollution manifest themselves, among other aspects, in the development or exacerbation of cardiovascular diseases including hypertension and stroke [27]. Hypertension, the most prevalent chronic disease in France, is a leading cause for cardiovascular, renal or cognitive complications. It is estimated that around 17 million people in France suffer from hypertension, with 6 million potentially unaware of their condition [24]. In addition to the screening and treatment of hypertension, it is critical to address the behavioural and environmental determinants of high blood pressure in a primary prevention perspective.

The impact of air pollution on blood pressure has been explored in several studies where positive associations between air pollutants such as NO_2_, O_3_, and particulate matter with a diameter of 2.5 µm or less (PM_2.5_) and high blood pressure have been reported [12, 32]. Most studies have primarily relied on exposure assessments of air pollutants computed using air dispersion models, land use regression models, or estimations from nearby air monitoring station [13, 16, 18]. However, these approaches typically do not capture the between-individual and temporal variations in terms of exposure to air pollutants in different outdoor and indoor micro-environments (including during the use of different modes of transport) [5]. In contrast, the present study took into consideration how exposures might vary across individuals and over time across microenvironments and transport modes, while using waist-worn accelerometers to control for the concomitant physical activity as a confounder [17].

In prior articles based on the first wave of the MobiliSense study, we found that a 1-μg/m^3^ increase in exposure to black carbon (BC) across 5 min was associated with a 0.57 mmHg and 0.36 mmHg increase in systolic and diastolic blood pressure respectively [6]. In a second study, we found that an increase in the exposure to a cocktail of air pollutants including BC, NO_2_, nitrogen monoxide (NO), carbon monoxide (CO) and O_3_ over the previous 5 min was associated with a 1.92 mmHg (95% CI: 0.63, 3.20) increase in systolic blood pressure [4]. However, these findings are based only on the first wave of the MobiliSense study. The present study exploit data from both the first and second waves of MobiliSense, which were between 1 and 2 years apart, with a particular focus on several potential sources of heterogeneity in the associations between short-term air pollutant exposure and ambulatory blood pressure.

In aim 1, we examined whether the blood pressure response to air pollutants was uniform across participants, or whether the slope of the relationship varied between participants. We also assessed whether this between-individual heterogeneity could be captured by specific individual characteristics (e.g. age, sex, body mass index, monthly alcohol consumption, physical activity level, and education level).

Prior studies have only focused on either short term or long-term effects of air pollutants. This study aimed to reconcile a short-term and longer-term vision by assessing whether and how short-term effects of air pollutants on blood pressure evolved over a period of 1–2 years [8]. Hence, in aim 2 we examined whether the sensitivity of blood pressure to air pollutant exposures remained constant or changed over time for participants (i.e., between wave 1 and wave 2 over a period of one or two years).

Only very few studies have investigated interactions of effects between air pollutants and temperature on blood pressure. A study in China reported significant interactions between temperature and particulate matter effects on SBP and DBP [34]. Heterogeneous temperature effects on SBP and DBP were also reported according to NO_2_. In another study [25, 26], higher concentrations of O_3_ resulted in stronger associations between outdoor temperature and cardiovascular mortality. To clarify this issue, in aim 3 we examined whether the impact of air pollution exposure on blood pressure was influenced by air temperature measurements, hypothesizing a stronger effect of air pollutants at higher temperature levels. This is especially critical to study in the context of climate change, particularly in urban areas, where extreme heat is projected to become more frequent.

## Methods

### Dataset, sampling, and participant selection

The MobiliSense study (funded by the European Research Council) was conducted in the Grand Paris region and included participants who were followed up in two waves, Wave 1 (2018–2020) and Wave 2 (2020–2022). Recruitment was carried out through a two-stage stratified random sampling technique, after the stratification of neighbourhoods by quartiles of area-level household income and quartiles of road traffic density. In the first stage, within each area income stratum, we randomly selected 30 neighbourhoods in each of the two extreme quartiles of traffic density (60 neighbourhoods in each area income quartile, i.e., 240 neighbourhoods overall). In the second stage, dwelling units from the chosen neighbourhoods were selected based on the census conducted by the National Institute of Statistics and Economic Studies in 2013–2014 [8]. Overall, 33,501 dwellings were selected in the 240 neighbourhoods. Participants had to be 30–64 years old on January 1, 2016. Eligible participants were contacted twice via postal mail to participate in the study. After complying with all the processes, 289 individuals accepted to participate in the first wave of the study and finalized the protocol. Informed consent was signed by all the participants prior to starting the study. Between 1 and 2 years after the first wave, 112 of the same participants agreed to participate in wave 2 and undergo the same sensor-based study as in wave 1 [8].

### Personal exposure to pollutants

#### Black carbon

BC was measured with an aethalometer (MicroAeth AE51, AethLabs, CA, USA). Participants wore the sensor for four days to measure BC concentrations at a 10 s time resolution. Measurements were adjusted according to time changes caused by daylight time savings in both waves and processed using the Optimised Noise Reduction Averaging algorithm to correct any high or low values caused by optical and electrical instruments. To prevent overloading of the filter used in the sensor and ensure that measurements were not underestimated by the sensor, filter changes were requested on day 2 at 8 pm when participants were recruited in winter. The Optimised Noise Reduction Averaging algorithm identified the changes in filter based on incremental light attenuation of at least 5 units between two data points. BC concentrations that remained constant for more than four hours despite participants switching microenvironments or negative for longer than 5 min were deleted from the analysis [5].

#### Gaseous pollutants and particulate matter

The personal air quality monitor (PAM) collected concentrations of NO_2_, NO, CO, and O_3_ at a 10 s resolution, and collected concentrations of PM_2.5_ at 1-min intervals. Details about the validity and reliability of this sensor in real time were published by the chemistry department of the University of Cambridge [9]. Under a scientific collaboration with them, calibration of three PAM sensors was carried out for a month at their laboratory. These devices were then used to calibrate the remaining PAM sensors used for this study. A device-specific calibration equation was designed to predict the corresponding concentrations of air pollutants mentioned above. Details about the calibration process and definition of equations are provided elsewhere [4]. The measurements were adjusted according to time changes caused by daylight time savings in both waves.

### Ambulatory blood pressure

Ambulatory systolic and diastolic blood pressure measurements were carried out every 30 min from wake time to bedtime in the first and third day of the study using an Arteriograph 24 ambulatory blood pressure monitor (TensioMed, Budapest, Hungary). The measurements were adjusted according to time changes caused by daylight time savings in both waves.

### Accelerometer

A wGT3X + tri-axial accelerometer was carried by the participants on the hip. It collected data in 1 s epochs. We used Actilife to calculate comprehensive information about sleep and physical activity [1]. We applied the so-called low frequency extension in the calculation to take into account slow movement of people. Physical activity was measured with the vector magnitude using Crouter algorithm in the Actilife application. This was calculated in 10 s epochs using raw accelerometer data, as follows:

$$\mathrm{VM}=\sqrt{\mathrm{Axis}\ {1}^{2}+\mathrm{Axis}\ {2}^{2}+\mathrm{Axis}\ {3}^{2}}$$

In Actilife, a value for the 3 axes being equal to a count of 0 for at least an hour along with spike tolerance of 2 min of non-zero epochs was set as non-wear and consecutively deleted from the data [6].

### Trip stages

GPS data were collected at a 5 s resolution using the BT-Q1000XT GPS receivers throughout the study. The data were processed using the Tripbuilder Web mapping application. Places visited by participants, the trips undertaken by participants, and the mode of transport used in each segment were all automatically identified with the application [7]. All this information was cross validated with the help of a mobility survey conducted with the participants over the phone to collect data on places visited and transport modes if they had been missed by the GPS receiver and/or algorithms. For this specific study, the proportions of time spent in different microenvironments such as home, places other than home, motorised transport, and non-motorised transport were calculated for intervals of 5 min to an hour preceding each blood pressure measurement [6].

### Meteorological measurements

The PAM monitor continuously recorded temperature and humidity (hereafter referred to as sensor-based temperature and humidity). Separately, the GPS receiver and mobility surveys continuously captured all the geographic locations. On the basis of each accurate geographic location assessed with spatial coordinates, hourly temperature data from Meteo France from the closest meteorological station were added for the duration of the follow up. Since the participant’s locations were not constant, the nearest station changed, and stations cannot be considered as comparable (indeed, one is located in a park while another is nearer from the traffic). In order to standardise and make more comparable the temperature and humidity measurements between the different stations, calculations of within-station z-scores of temperature were carried out for all the stations for the whole study period. The hourly temperature z-score from the nearest station was assigned to each participant’s GPS and location point. Misclassification was accounted for by discarding the temperature and humidity exposures from points that were > 15 km away from the nearest station [6]. Thus the present study had access to both sensor-based temperature and station-based temperature.

### Sociodemographic covariates

Additional covariates were age, sex, body mass index (BMI), physical activity status, medication against hypertension, monthly alcohol intake, employment level, education level, residence, wave indicator, week day, and time of the day. The sociodemographic and behavioural information was derived from a standard computerised questionnaire, while BMI was derived from measured height and weight [8]. Age, BMI, and monthly alcohol intake were treated as continuous variables. Of note, the age variable was calculated as the difference between the start time of the monitoring period and the birth date, and so was different for wave 1 and wave 2 (as participants were 1–2 years older in the second wave). Sex was coded as females vs. males. Physical activity status was coded as a binary variable, where people who undertook any regular physical activity were assigned as “yes” and the rest were assigned to “no”, based on a questionnaire. Medication against hypertension was divided into three categories: participants with no hypertension, those with hypertension who took regular medication, and those with hypertension who did not take medication. Three categories were used to code education level: equal to baccalaureate, higher than baccalaureate, and lower than baccalaureate. Employment level was assigned to five categories: stable job, unstable job, unemployment, retired, and other. Residence had three categories: Paris, close suburbs (adjacent counties to Paris), and far suburbs (non-adjacent counties). Alcohol intake was self-reported as the number of drinks per day, week, month, or year, and was calculated as monthly alcohol consumption [4]. We differentiated between weekday and weekend, whereas the time of the day was divided into three categories: morning (6 am to < 12 noon), evening (12 noon to 6 pm), and night (6 pm to < 6 am). Wave indicator was coded with wave 1 as 0 and wave 2 as 1.

### Statistical analysis

#### Analytical sample

Verification of non-wear time for the different sensors was carried out based on the reports of the participants for all the sensors and deleted prior to the final processing. The non-wear time for the accelerometer was calculated automatically with Actilife and deleted prior to processing. The final analytical database was set by appending the samples from both waves, with many participants (but not all) present in the two waves. The analytical database only included blood pressure measurements from the first day of the study (where the two air pollutant monitors and the blood pressure monitor were carried by the participants). It included 4354 blood pressure measurements (3319 observations from wave 1 and 1035 observations from wave 2) for 235 participants who were present in either wave 1 or wave 2. Among the 235 participants, there were 166 participants present solely in wave 1 whereas 55 participants were present in both waves. Additionally, there were 14 participants present in wave 2 who were not retained in wave 1, due to missing data.

#### Overall analytical strategy

Short durations of exposure to pollutant concentrations (only 5 min averages prior to blood pressure measurements) were used in this study as larger association with ambulatory blood pressure was found at shorter exposure windows [6]. For the blood pressure measurements, only the data from day 1 were used, as there were no corresponding gaseous exposure measurements from the PAM sensors in day 3. All the time varying factors (short-term air pollutant averaged concentrations, physical activity status, sensor temperature and relative humidity, and proportion of time spent in different contexts such as home, motorized vehicle, and non-motorized vehicle) were calculated across 5-min windows prior to blood pressure measurements. The z-scores of temperature derived from Meteo France stations were averaged over the 30 min before each blood pressure measurements.

Linear mixed models were used to examine the association between air pollutants and blood pressure. All the mixed models controlled for all the air pollutants, time-invariant factors (age, sex, BMI, education, employment, monthly alcohol consumption, medication against hypertension, residence area, day of the week or weekday, and wave indicator) and time-varying factors. A continuous autoregressive correlation structure was specified in all the models to capture temporal autocorrelation within individuals over time. In order to account for possible multicollinearity issues with inclusion of multiple air pollutants in the same models, the variance inflation factor (VIF) was calculated for all of the models; no such issue was detected. As our baseline model, the mixed linear model only included an individual-level random intercept, the variables listed above, and a temporal autocorrelation structure (phi). The linear mixed effect models were then complexified according to the different objectives listed below.

#### Heterogeneity between participants

In order to estimate the heterogeneity in the association of air pollutants with blood pressure, we fitted random coefficient models, to allow the effect of air pollutants to vary between individuals. As a parsimonious modelling strategy, we only included a random coefficient for an air pollutant if it led to a better fit of the model to the data, as assessed with the Akaike Information Criterion (AIC) (as explained in detail in Additional file: Table 2). With this model, the air pollutants with substantial heterogeneity in their association with blood pressure were identified, which is also of interest.

In a second step, interaction terms were included in the random coefficient model that was selected to test for heterogeneity in the air pollutant – blood pressure associations according to specific individual variables (age, sex, BMI, physical activity, education, and monthly alcohol consumption). Interaction terms were retained if they were associated with a p-value < 0.01, to detect differences between groups of major importance.

#### Heterogeneity over time

In a separate set of models only including participants present in both waves (*n =* 55), we then tested for heterogeneity in the air pollutant effects on blood pressure over time (i.e., from wave 1 to wave 2), while considering that the air pollution effect could vary between individuals and that the change over time in the air pollution effect could also vary between individuals. Separately for each pollutant, the fully adjusted linear model included: the effect of the air pollutant of interest (also adjusted for other air pollutants), a random coefficient for the air pollutant of interest, an interaction between this air pollutant and the wave indicator, and finally a random coefficient for the latter interaction term. In addition, a sensitivity analysis was conducted to assess whether the association between each air pollutant and blood pressure changed from wave 1 to wave 2 in a fixed effect model, estimated by controlling for one dummy variable related to each participant (while the participant-level random effects were removed from the model). The fixed effect models were adjusted for all the air pollutants and time varying covariates.

#### Heterogeneity according to temperature

In a separate set of models, using an interaction term added to the fully adjusted regression model (only comprising a random intercept), we finally examined whether air pollutant effects on blood pressure varied according to air temperature (separately for each air pollutant and for the sensor-measured temperature and the temperature z-scores determined from the Meteo France station data). We descriptively explored the relationship between air pollutants and temperature with a boxplot. Using the coefficients of the fully adjusted random intercept regression model including an interaction term between the air pollutant effect and temperature, we predicted the estimate of association between BC and ambulatory blood pressure at the different deciles of temperature. As sensitivity analyses, interactions of effects between temperature and air pollutants were also tested in the random coefficient model that was selected in the previous step, and in a fixed effect model. The random coefficient model was adjusted for all the air pollutants, time varying covariates, and time invariant covariates, whereas the fixed effect model was only adjusted for the air pollutants and the time varying covariates.

## Results

### Descriptive information

In the sample of 235 participants, the mean age was 50.3 years, and the mean BMI was 25. Amongst the sample, 55% were female and 45% were male. Approximately 76.2% of the participants were living in sub-urban areas close to Paris, 23% were living in Paris, and the rest lived in far-suburban areas. In terms of education and employment levels, the majority of participants (70.6%) had a higher diploma than baccalaureate and 66.8% of the participants had a stable job (see Additional file: Table 1 for additional descriptive information). A logistic model for the 235 participants with a binary outcome variable corresponding to being present in wave 2 showed that participants present in wave 2 were not different than participants in wave 1 based on the following individual variables: age, sex, residence, BMI, employment level, education level, household income, medication against hypertension, and monthly alcohol intake.

The average systolic and diastolic blood pressure in wave 1 (among 221 participants) were 126.4 mmHg and 72.5 mmHg respectively. The corresponding values in wave 2 (for 69 participants) were 125.5 mmHg and 72.0 mmHg respectively.

The average concentrations of air pollutants in wave 1 over the 5 min before blood pressure measurement were 1.13 μg/m^3^ for BC, 22.0 ppb (parts per billion) for NO, 11.6 ppb for NO_2_, 831 ppb for CO, and 16.3 ppb for O_3_. The corresponding figures in wave 2 were 0.99 μg/m^3^ for BC, 21.6 ppb for NO, 12.6 ppb for NO_2_, 1368 ppb for CO, and 14.9 μg/m^3^ for O_3._

### Baseline models

Adjusted estimates for the associations of the different air pollutants over the previous 5 min with blood pressure in the baseline random intercept model without random coefficients are reported in Table 1. Of note, although we report coefficients for all the covariates in the models, the readers should mostly focus their attention on the main target estimates, i.e., those for the 5 air pollutants. After adjustment for sociodemographic variables and other air pollutants, a 1 μg/m^3^ increase in BC concentration was associated with an increase of 0.64 mmHg (95% CI: 0.29, 0.99) in systolic blood pressure and 0.18 mmHg (-0.11, 0.48) in diastolic blood pressure (the confidence interval crossed the null for diastolic blood pressure). In the same model, a 10 ppb increase in NO_2_ was associated with an increase by 0.60 mmHg (0.11, 1.08) in diastolic blood pressure, while a 10 ppb increase in O_3_ was associated with an increase of 1.44 mmHg (0.78, 2.09) in systolic blood pressure and 0.64 mmHg (0.10, 1.19) in diastolic blood pressure. In this model where air pollutants were adjusted for each other, we did not observe any marked association of NO and CO with blood pressure.

**Table 1:** Fully adjusted associations of air pollutants with systolic and diastolic blood pressure in a random intercept regression model (all air pollutants included in the same model)^a^

Variables	Systolic blood pressure	Diastolic blood pressure
Air pollutants		
BC (unit: µg/m^3^)	0.64 (0.29, 0.99)	0.18 (-0.11, 0.48)
NO (unit: 10 ppb)	-0.07 (-0.19, 0.05)	-0.01 (-0.12, 0.09)
NO_2_ (unit: 10 ppb)	0.47 (-0.11, 1.05)	0.60 (0.11, 1.08)
CO (unit: 100 ppb)	0.01 (-0.03, 0.04)	-0.03 (-0.06, -0.01)
O_3_ (unit: 10 ppb)	1.44 (0.78, 2.09)	0.64 (0.10, 1.19)
Sex (vs. female)		
Male	8.74 (5.57, 11.91)	8.67 (5.99, 11.35)
Age (years)	0.47 (0.26, 0.68)	0.32 (0.15, 0.50)
Body mass index	0.04 (-0.13, 0.21)	0.02 (-0.12, 0.16)
Physical activity (vs. no)		
Yes	-2.16 (-4.67, 0.36)	-2.10 (-4.17, -0.02)
Monthly alcohol consumption (units)	-0.05 (-0.17, 0.08)	-0.03 (-0.13, 0.07)
Education (vs. higher than high school)		
Equal to high school	-0.93 (-4.37, 2.51)	-0.90 (-3.78, 1.98)
Lower than high school	-1.14 (-7.74, 5.46)	-1.51 (-7.09, 4.07)
Residence (vs. far suburbs)		
Close suburbs	-1.81 (-8.28, 4.65)	1.11 (-4.13, 6.35)
Paris	-3.44 (-10.76, 3.88)	1.07 (-4.93, 7.07)
Medication against hypertension (vs. no hypertension)		
Not medicated	17.52 (8.03, 27.0)	14.92 (7.00, 22.85)
Regularly medicated	3.76 (-2.25, 9.76)	2.11 (-2.95, 7.18)
Employment (vs. stable employment)		
Retired	-2.08 (-6.73, 2.56)	-0.51 (-4.38, 3.37)
Unemployed	6.75 (0.83, 12.66)	3.90 (-0.97, 8.78)
Unstable employment	0.75 (-3.75, 5.26)	0.79 (-2.93, 4.51)
Other employment	-2.75 (-6.32, 0.81)	-2.50 (-5.47, 0.47)
Proportion of time at domicile	-2.32 (-3.49, -1.14)	-2.58 (-3.55, -1.61)
Proportion of time in motorized transport	0.16 (-2.22, 2.53)	-2.51 (-4.52, -0.50)
Proportion of time in non-motorized transport	0.35 (-2.20, 2.90)	-0.29 (-2.45, 1.87)
Accelerometer vector magnitude	0.02 (0.01, 0.02)	0.00 (-0.00, 0.01)
Sensor temperature	-0.16 (-0.36, 0.05)	-0.06 (-0.24, 0.11)
Sensor humidity	0.08 (-0.01, 0.16)	0.05 (-0.02, 0.12)
Station-measured temperature	-1.56 (-2.35, -0.76)	-0.78 (-1.43, -0.13)
Wave 2 (vs. wave 1)	0.36 (-1.08, 1.81)	0.10 (-1.07, 1.27)
Time of the day (vs. afternoon)		
Evening	0.85 (-0.12, 1.83)	0.69 (-0.11, 1.50)
Morning	-0.97 (-2.16, 0.22)	0.30 (-0.68, 1.28)
Day of the week (vs. weekday)		
Weekend	-3.35 (-5.43, -1.28)	-1.93 (-3.64, -0.23)
Random intercept standard deviation	10.86	9.24

MobiliSense study, 235 participants, 4354 blood pressure measurements

*CI* Confidence interval

^a^The Akaike information criterion (AIC) for the systolic model is 33,853.32 and for the diastolic model is 32,404.38

Of note, when the different air pollutants variables were included each in a separate random intercept model adjusted for other covariates (one air pollutant variable per model as reported in Additional file: Table 7), a 1 μg/m^3^ increase in BC concentration was associated with an increase of 0.63 mmHg (0.30, 0.97) and a 10 ppb increase in O_3_ was associated with a 1.21 mmHg increase (0.06, 1.82) in systolic blood pressure. In the models for diastolic blood pressure, only a negative association with CO was noted.

### Heterogeneity between participants

As shown in Appendix Table 2, in models controlling for all air pollutants and time-invariant and time-varying covariables, we found that adding to the models 3 participant-level random coefficients for the effects of NO, NO_2_, and O_3_ led to the best fit to the data (as shown with the AIC), for both systolic and diastolic blood pressure. The AIC did not lead us to include a random coefficient for the other two air pollutants (BC and CO).

Table 2 represents the associations estimated from the fully-adjusted random coefficient models (i.e., with NO, NO_2_, and O_3_ as random coefficients) between air pollution exposures and ambulatory blood pressure. A unit increase in BC was associated on average with an increase of 0.55 mmHg in systolic blood pressure (0.19, 0.91). Similarly, a 10 ppb increase in NO_2_ exposure on average increased diastolic blood pressure by 0.80 mmHg (0.07, 1.53). Considering the main effect of the pollutant and the between-individual random coefficients, a 10 ppb increase in NO was associated with an increase in systolic blood pressure ranging from -0.15 to 0.41 mmHg between individuals and with an increase in diastolic blood pressure ranging from -0.52 to 0.43 mmHg, corresponding to the 10th and 90th percentiles of the effects across all individuals as calculated from the random coefficient. The corresponding effects for a 10 ppb increase in NO_2_ were from -1.62 to 2.90 mmHg between individuals for systolic blood pressure and from -0.81 to 2.57 mmHg for diastolic blood pressure. The corresponding effects ranged from -4.09 to 5.61 mmHg and from -2.19 to 3.14 mmHg for a 10 ppb increase in O_3_.

**Table 2:** Fully adjusted associations of air pollutants with systolic and diastolic blood pressure in a random coefficient regression model (all air pollutants included in the same model)^a^

Variables	Systolic blood pressure	Diastolic blood pressure
Air pollutants		
BC (unit: µg/m^3^)	0.55 (0.19, 0.91)	0.11 (-0.20, 0.42)
NO (unit: 10 ppb)	0.13 (-0.09, 0.35)	-0.02 (-0.22, 0.17)
NO_2_ (unit: 10 ppb)	0.73 (-0.21, 1.67)	0.80 (0.07, 1.53)
CO (unit: 100 ppb)	-0.00 (-0.04, 0.03)	-0.03 (-0.06, -0.00)
O_3_ (unit: 10 ppb)	0.93 (-0.51, 2.37)	0.41 (-0.48, 1.31)
Random intercept standard deviation	19.50	12.56
Random slopes standard deviation		
NO_2_	3.92	2.72
NO	0.62	0.72
O_3_	7.73	3.73
Residual standard deviation	10.48	9.11

MobiliSense study, 235 participants, 4354 blood pressure measurements

The two models included the following variables: short-term exposure to all the air pollutants (except PM_2.5_), accelerometer vector magnitude, physical activity status, temperature (sensor and station based), relative humidity (measured by sensor), proportion of time spent in different contexts (home, motorized vehicle, and non-motorized vehicles), wave indicator, time of the day, day of the week, residence area, age, sex, education, employment, monthly alcohol consumption, body mass index, and medication against hypertension

CI: confidence interval

^a^The Akaike information criterion (AIC) for the systolic model is 33,595.45 and for diastolic model is 32,325.83

As shown in the comparison of Tables 1 and 2, the association between O_3_ and blood pressure was lost after inclusion of the random coefficients, in particular the random coefficient for O_3_. It suggests that the association between O_3_ and blood pressure is of very diverse magnitude between the individuals of the sample and that most likely O_3_ is not associated with blood pressure for all participants.

When we assessed how the association between air pollutants and blood pressure varied across different sociodemographic groups, we found that the association between NO and systolic blood pressure differed by sex. Specifically, the association between NO and systolic blood pressure was weaker in males (-0.40, 95% CI: -0.78, -0.02) compared to females. The interaction between O_3_ and monthly alcohol consumption revealed that for every 1-unit increase in monthly alcohol consumption, the elevation in systolic blood pressure associated with a 10 ppb increase in O_3_ was reduced by 0.12 mmHg (-0.23, -0.01). The average monthly alcohol consumption was 9.36 drinks per month (range: 0–61). However, 75% of the participants reported consuming less than 13 drinks per month.

### Heterogeneity over time

We found evidence of variation across waves in the association between O_3_ and systolic blood pressure, as suggested by the interaction term between O_3_ and the wave indicator in a simple random intercept model (Additional file: Table 3). A more complex random coefficient model including an interaction term between O_3_ and wave, and two random coefficients for the O_3_ concentration and for the O_3_ – wave interaction was found to have an AIC of 13,308.79, compared to an AIC of 13,516.84 for the random intercept model with an interaction between O3 and wave shown in Additional file: Table 3. As the association between O_3_ and systolic blood pressure is difficult to quantify in our complex random coefficient model, we provide the distribution of the calculated strength of association across individuals in waves 1 and 2 in Table 3.

**Table 3:** Distribution of the association between a 10 ppb increase in O_3_ and systolic blood pressure across individuals in wave 1 and wave 2 from a random coefficient model^a^

Measures	Effect of O_3_ in wave 1	Effect of O_3_ in wave 2
25^th^ quartile	-1.34	-0.22
Mean	3.67	1.28
Median	3.12	1.54
75^th^ quartile	6.34	2.67

MobiliSense study, 55 participants, 1726 blood pressure measurements

^a^The model included the following variables: short-term exposure to all the air pollutants (except PM2.5), accelerometer vector magnitude, physical activity status, temperature (sensor and station based), relative humidity (measured by sensor), proportion of time spent in different contexts (home, motorized vehicle, and non-motorized vehicles), wave indicator, time of the day, day of the week, residence area, age, sex, education, employment, monthly alcohol consumption, body mass index, and medication against hypertension

The association between O_3_ and systolic blood pressure was found to be lower in wave 2 than in wave 1 across the whole distribution. This findings was further confirmed in a sensitivity analysis where the association was assessed with a fixed effects model (within-participant comparison by controlling for a dummy variable for each individual). In this model, an interaction indicated that the association between O_3_ and systolic blood pressure was weaker in wave 2 (-2.29, 95% CI: -3.72, -0.85) compared to wave 1 (as shown in Additional file: Table 4).

### Heterogeneity according to temperature

To explore the modifying effect of temperature on the air pollution – blood pressure association, we first introduced the interaction of either sensor-measured temperature or station-measured temperature with each air pollutant in the baseline random intercept model mentioned in Table 1. We found an interaction between BC and station-measured temperature for both systolic blood pressure (*p*-value = 0.001, reduction in AIC = 9.9) and diastolic blood pressure (*p*-value = 0.02, reduction in AIC = 3.5). Sensor temperature was not found to interact with air pollutants.

**Fig. 1:**
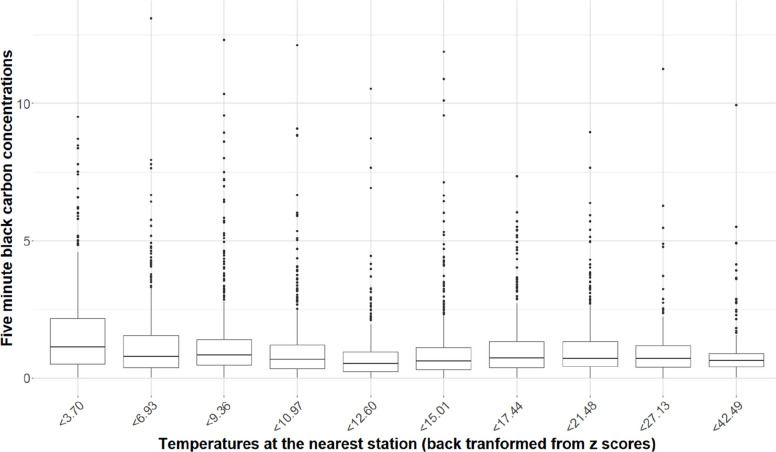
Distribution of black carbon across deciles of outdoor temperature in MobiliSense study (235 participants, 4354 blood pressure measurements). In the boxplot, the station-measured temperature is provided as deciles of temperature recorded in Meteo France stations. The temperatures represented in Fig. 1 have been back transformed from the z-scores calculated for each station included in the MobiliSense study. The corresponding z-score values for the deciles ranged from -2.1 to 3.7

As descriptive information, Fig. 1 shows the distribution of BC over the previous 5 min before blood pressure measurement across different deciles of station-measured temperature. The Jonckheere-Terpstra trend test statistic (z = -7.07) was negative with a p-value far below 0.05 (5*10^–12^), which is related to the fact that BC was at its highest levels for the lowest deciles of temperature.

**Fig. 2:**
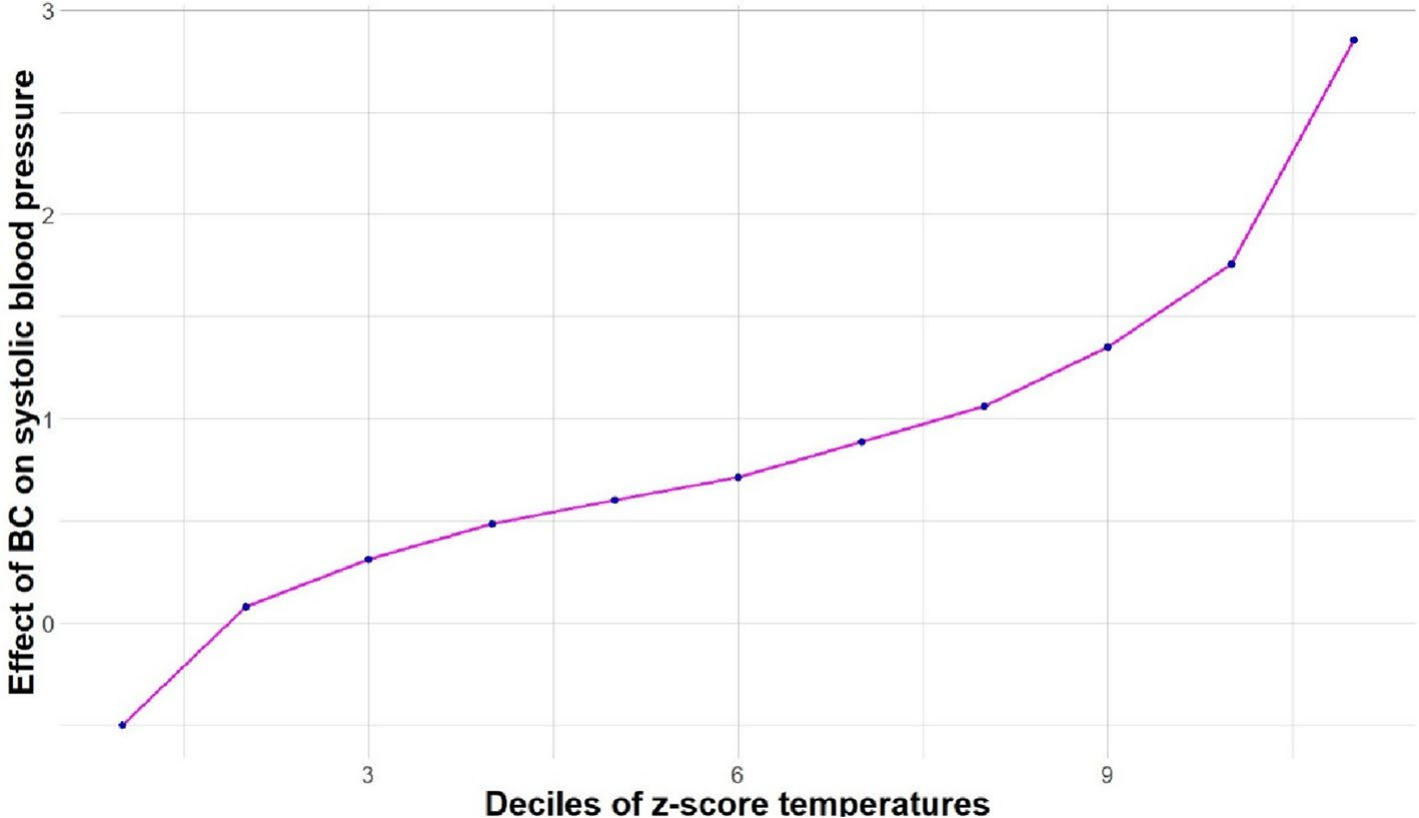
Prediction of the slope of the BC (µg/m^3^) – systolic blood pressure association according to the decile of station-measured temperature z-scores in MobiliSense study (235 participants, 4354 blood pressure measurements). The estimated random intercept model was used to predict the effect of BC on systolic blood pressure according to the deciles of station-measured temperature z-scores. This model included the following variables: short-term exposure to all the air pollutants (except PM_2.5_), accelerometer vector magnitude, physical activity status, temperature (sensor and temperature from Meteo France), an interaction term between BC and station-measured temperature, relative humidity (measured by sensor), proportion of time spent in different contexts (home, motorized vehicle, and non-motorized vehicles), wave indicator, time of the day, day of the week, residence area, age, sex, education, employment, monthly alcohol consumption, body mass index, and medication against hypertension

**Fig. 3:**
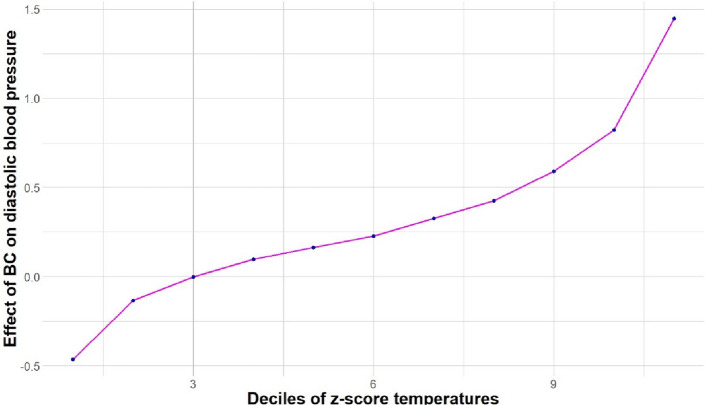
Prediction of the slope of the BC (µg/m^3^) – diastolic blood pressure association according to the decile of station-measured temperature z-scores in MobiliSense study (235 participants, 4354 blood pressure measurements). The estimated random intercept model was used to predict the effect of BC on diastolic blood pressure according to the deciles of station-measured temperature z-scores. This model included the following variables: short-term exposure to all the air pollutants (except PM2.5), accelerometer vector magnitude, physical activity status, temperature (sensor and temperature from Meteo France), an interaction term between BC and station-measured temperature, relative humidity (measured by sensor), proportion of time spent in different contexts (home, motorized vehicle and non-motorized vehicles), wave indicator, time of the day, day of the week, residence area, age, sex, education, employment, monthly alcohol consumption, body mass index, and medication against hypertension

Figures 2 and 3 present the association between BC and systolic and diastolic blood pressure predicted from the model for the deciles of the z-score of station-measured temperatures. The association between BC and both systolic and diastolic blood pressure was stronger at higher outdoor temperature levels. The values of the predicted associations and confidence intervals are shown in Additional files: Table 8.

As a sensitivity analysis, we replicated this analysis using a fixed-effect model (controlling for one dummy variable for each participant). The interaction between BC and station-measured temperature was also observed for both systolic and diastolic blood pressure in such fixed effect models (as shown in Additional file: Table 5). The association between BC and systolic or diastolic blood pressure was stronger at higher outdoor temperature levels (+ 0.64, 95% CI: 0.32, 0.96 and + 0.34, 95% CI: 0.07, 0.61, respectively, for each 1-unit increase in the temperature z-score).

As a second sensitivity analysis, we reestimated how the association between BC and blood pressure differed according to the temperature level using the random coefficient model that was selected above, instead of the random intercept model. As shown in Additional file: Table 6, with a such a model, the association between BC and blood pressure was found to be stronger at higher temperature levels for diastolic blood pressure but not for systolic blood pressure.

## Discussion

We found that the association between multiple air pollutants and blood pressure varied in magnitude between sociodemographic groups. The association between O_3_ and systolic blood pressure was attenuated over the course of two waves. We also documented a stronger positive association between BC and blood pressure at higher levels of outdoor temperature.

In the initial model considering simultaneously the different air pollutants, we found that both a higher concentration of BC and a higher concentration of O_3_ were associated with a higher systolic blood pressure, while higher concentrations of NO_2_ and O_3_ were associated with a higher diastolic blood pressure.

Several studies have found that the long-term exposure to higher concentrations of O_3_ was associated with the incidence of hypertension [11, 17]. Moreover, there has been shorter-term effect studies. For example, a South-Korean study reported that an interquartile range increase in O_3_ was associated with an increase in systolic blood pressure by 0.55 (± 0.14) mmHg at 3–5 lag hours [10]. However, another study reported that an increase in O_3_ concentrations reduced systolic blood pressure with a lag of 0–14 days [20].

We found that O_3_ had a reduced association with systolic blood pressure in people who drink alcohol. A study in China reported similar findings where negative associations between O_3_ and blood pressure were more evident in former or current alcohol drinkers [30].

We also found that the association of NO with systolic blood pressure was higher in women. Previous studies have documented that blood pressure regulation and development are dependent on biological effects of sex hormones and reproductive events [3, 14]. Several studies demonstrated blood pressure trajectories over time where males tend to have higher systolic and diastolic blood pressure than females until 30 years of age, after which females have a steeper rise throughout their life [23, 28]. It provides evidence for how females might develop risks for cardiovascular complications even at lower initial levels of blood pressure [19, 22]. Additionally, due to socially constructed gender roles, women may be more exposed to air pollutants as for example they are more generally responsible for household chores, and this could increase their susceptibility to air pollutants [21].

The strength of the association between O_3_ concentrations and blood pressure was found to decrease over time. It should be emphasized that the second wave of MobiliSense started on March 5 2020 and lasted until June 2022. Although no data were collected during the stricter COVID lockdown period from March 17 2020 to May 11 2020, the whole data collection of the second wave occurred in a period that was perturbated by COVID where restrictions in mobility or higher uptake of telework could result in lower traffic and so lower O_3_ pollution. As a potential explanation, a nonlinear association between O_3_ and blood pressure could result in a weaker association in wave 2 at lower overall levels of O_3_.

In this study, we observed that the strength of the association between BC and blood pressure increased with outdoor station-measured temperature. Of note, such an interaction was not documented with sensor-measured temperature. It should be kept in mind that station-measured temperature and sensor temperature reflect different things. The sensor-based temperature is a mix of indoor and outdoor temperature depending on whether the participant is indoor or outdoor. Differently, station-measured temperature reflects outdoor temperature. Only few past studies have reported an interaction of effects between the exposure to air pollutants and temperature in relation to blood pressure; indeed most studies that considered both air pollutant and temperature effects on blood pressure estimated their independent association with the outcome [15]. A study from China reported interactions between the effects of temperature and particulate matter on systolic and diastolic blood pressure; the effect of temperature was found to be higher at higher particulate matter levels [34]. We interpret the stronger association between BC and blood pressure observed at higher temperature levels in our study as resulting from a cumulation of two exposures.

### Strengths and limitations

Several strengths exist in this study. One of the major strengths was the use of multiple wearable sensors to measure air pollution, temperature, and blood pressure dynamically, which reduces exposure and outcome misclassification. Air pollution and temperature misclassification is often a limitation in studies that extract data from stationary monitoring stations. In such studies, exposure misclassification could lead to underestimation of associations [29]. Another highlight of the study is the high temporal resolution of the MobiliSense data, allowing for short-term acute impacts of air pollution (e.g., in the 5 previous minutes) to be assessed. The consistent monitoring over time of the various dimensions was helpful in trying to model causal relationships between the exposures and blood pressure after accounting for time-varying confounders [4]. In addition, the advanced random intercept and random coefficient models that we used made it feasible to account for complex patterns of heterogeneity present amongst the individuals and across the two waves. Finally, a strength of our analytical methodology was that we compared random effect models with fixed effect models, which confirmed the observed findings on a within-participant comparison basis.

We acknowledge the limitations and challenges we faced in this study. The sensors used in this study measured different pollutants across different days (not all pollutants on every day). As a result, this particular analysis could be carried out for day 1 only, as there was no information for gaseous pollutants on day 3, thus limiting the number of days included in the study. Additionally, the sample size of the study at the participant level was relatively small, especially due to the significant drop out between wave 1 and wave 2 attributable to the important burden of the protocol. This relatively low sample size contributed to the often large standard errors in the models. It also suggests that caution is needed to generalize the findings to whole background population of Grand Paris.

## Conclusion

First, this study provides novel evidence on how the relationship between air pollution exposure and the blood pressure response may vary across different dimensions, including individual profiles, time, and temperature. The differentiated influence of diverse air pollutants on blood pressure demonstrated the vulnerability of certain population groups over others, which needs to be accounted for in the management of the hypertensive disease in these groups if further validated. Similarly, as an intriguing finding, we observed that the strength of the O_3_ – blood pressure association decreased over time. Finally, we found that the association between BC and blood pressure was stronger at higher temperature levels. In the context of the growing temperature in France and more frequent occurrence of heat waves as a result of climate change, this finding underscores the need to reform or improve urban and transport systems, because in the urban heat islands of cities people are exposed to both BC emitted by motor vehicles and to particularly high temperatures during heatwaves.

## Supplementary Information

Supplementary Material 1.

## Data Availability

The datasets analysed during this study are not publicly available to maintain the confidentiality of participants.
