# Supplementary material for: Investigation of sociodemographic, temporal, and meteorological heterogeneity in the short-term blood pressure response to air pollutants
Source: Environ Health. 2026 Feb 28;25:27. doi: 10.1186/s12940-026-01275-x (PMC13059608; doi:10.1186/s12940-026-01275-x)
Supplement: Supplementary file 1 — Supplementary Material 1. [file 12940_2026_1275_MOESM1_ESM.docx]

Additional file

| Table 1: Descriptive statistics for time-variant and invariant characteristics of participants | |
| --- | --- |
| Variables | Mean (SD) or N (%) |
| Age (years) | 50.3 (8.7) |
| Sex |  |
| Female | 130 (55.4%) |
| Male | 105 (44.6%) |
| Body mass index | 25.2 (6.1) |
| Physical activity |  |
| Yes | 177 (75.3%) |
| No | 58 (24.7%) |
| Monthly alcohol consumption (units) | 9.1 (9.8) |
| Education |  |
| Higher than high school | 166 (70.6%) |
| Equal to high school | 54 (23.0%) |
| Lower than high school | 15 (6.4%) |
| Residence |  |
| Paris | 54 (23.0%) |
| Close suburbs | 179 (76.2%) |
| Far suburbs | 2 (0.8%) |
| Medication against hypertension |  |
| Not medicated | 4 (1.7%) |
| Regularly medicated | 16 (6.8%) |
| Not having hypertension | 215 (91.5%) |
| Employment |  |
| Stable employment | 157 (66.8%) |
| Retired | 27 (11.5%) |
| Unemployed | 7 (3.0%) |
| Unstable employment | 13 (5.5%) |
| Other employment | 31 (13.2%) |
| MobiliSense study, 235 participants, 4354 blood pressure measurements | |

Table 1 reports the descriptive statistics of the participants in the MobiliSense study. In the sample of 235 participants, the mean age was 51 years, and the mean BMI was 25. Amongst the sample, 55% were female and 45% were male. Approximately 76.2% of the participants were living in sub-urban areas close to Paris, 23% were living in Paris, and the rest lived in far-suburban areas. In terms of education level, the majority of participants (70.6%) had a higher diploma than baccalaureate, 23% had a baccalaureate diploma, and 6.4% of the participants had educational qualifications lower than Baccalaureate. At the time of the study, 66.8% of the participants had a stable job, 5.5% had an unstable job, 3% were unemployed, and 11.5% were retired. About 91.5% of participants did not report having hypertension, while the remaining participants were either taking regular medication against hypertension (6.8%) or no medication at all while being aware of having hypertension (1.7%).

| Table 2: Goodness of fit expressed with AICs of models comprising one, two, or three random coefficients for air pollutants^1^ | | |
| --- | --- | --- |
| Pollutants | Systolic blood pressure (AIC) | Diastolic blood pressure (AIC) |
| One coefficient |  |  |
| BC | 33806.9 | 32393.6 |
| NO | 33829.6 | 32382.6 |
| NO_2_ | 33838.0 | 32391.3 |
| CO | 33856.4 | 32400.8 |
| O_3_ | 33658.7 | 32366.7 |
|  |  |  |
| Two coefficients |  |  |
| BC+NO | 33768.6 | 32369.8 |
| BC+NO_2_ | 33800.6 | 32383.0. |
| BC+O_3_ | 33638.6 | 32361.3 |
| NO+NO_2_ | 33815.5 | 32369.8 |
| NO+O_3_ | 33645.8 | 32347.0 |
| NO_2_+O_3_ | 33605.4 | 32354.5 |
|  |  |  |
| Three coefficients |  |  |
| BC+NO+NO_2_ | 33769.0 | 32356.7 |
| BC+NO_2_+O_3_ | 33607.9 | 32353.3 |
| BC+NO+O_3_ | 33625.6 | 32341.2 |
| NO+NO_2_+O_3_ | 33595.5 | 32325.8 |
|  |  |  |
| Final model |  |  |
| NO+NO_2_+O_3_ | 33595.5 | 32325.8 |
| MobiliSense study, 235 participants, 4354 blood pressure measurements  ^1^All models included the following variables: short-term exposure to all the air pollutants (except PM_2.5_), accelerometer vector magnitude, physical activity status, temperature (sensor and station based), relative humidity (measured by sensor), proportion of time spent in different contexts (home, motorized vehicle, and non-motorized vehicles), wave indicator, time of the day, day of the week, residence area, age, sex, education, employment, monthly alcohol consumption, body mass index, and medication against hypertension. | | |

Table 2 provides the AIC values for models adjusted for individual characteristics and time-varying covariates, including coefficients for all the air pollutants, and specifying random coefficients for one, two, or three air pollutants simultaneously to assess whether their effect size vary between individuals. Akaike information criterion (AIC) values were compared to determine the model with the best fit according to complexity.

Random coefficient models were estimated with only one random coefficient at a time and the AIC values were recorded to assess whether the model had a better fit compared to the model with no random coefficient. A reduction in AIC by at least 3 units was considered as a better fit compared to higher AICs (Bista et al., 2022). We gradually introduced random coefficients for two air pollutants step by step (e.g., BC+NO, BC+NO_2_, etc.) to assess the mutually adjusted between-individual variability in their effect, and ultimately random coefficients for 3 air pollutants to determine the model that best accounts for variability across different individuals. We chose the multilevel model that minimized the AIC, with the best compromise of model fit to the data and parsimony. In both systolic and diastolic blood pressure models, random coefficient models with NO, NO_2_, and O_3_ as random coefficients were found to have the best model fit.

| Table 3: Interaction of effects between O_3_ and wave for systolic blood pressure, identified from a random intercept model^1^ | |
| --- | --- |
| Variables | Systolic blood pressure |
| Air pollutants |  |
| BC (unit: µg/m^3^) | 1.19 (0.62, 1.76) |
| NO (unit: 10 ppb) | -0.24 (-0.52, 0.03) |
| NO_2_ (unit: 10 ppb) | 0.48 (-0.48, 1.44) |
| CO (unit: 100 ppb) | 0.04 (-0.01, 0.08) |
| O_3_ (unit: 10 ppb) | 2.73 (1.43, 4.04) |
| Sex (vs. female) |  |
| Male | 5.59 (-0.27, 11.46) |
| Age (years) | 0.41 (0.03, 0.78) |
| Body mass index | -0.19 (-0.41, 0.04) |
| Physical activity (vs. no) |  |
| Yes | -3.01 (-6.56, 0.55) |
| Monthly alcohol consumption (units) | -0.14 (-0.32, 0.03) |
| Education (vs. higher than high school) |  |
| Equal to high school | -0.13 (-6.05, 5.79) |
| Lower than high school | 18.09 (2.86, 33.32) |
| Residence (vs. far suburbs) |  |
| Close suburbs | 0.55 (-6.66, 7.75) |
| Paris | 3.73 (-5.75, 13.22) |
| Medication against  hypertension (vs. no hypertension) |  |
| Not medicated | 29.06 (16.07, 42.05) |
| Regularly medicated | 12.06 (2.16, 21.96) |
| Employment (vs. stable employment) |  |
| Retired | -2.28 (-8.79, 4.23) |
| Unemployed | 9.11 (0.22, 17.99) |
| Unstable employment | 4.43 (-1.80, 10.66) |
| Other employment | 0.95 (-4.33, 6.23) |
| Proportion of time at domicile | -1.21 (-3.22, 0.80) |
| Proportion of time in motorized transport | 0.31 (-3.81, 4.42) |
| Proportion of time in non-motorized transport | 0.09 (-4.41, 4.60) |
| Accelerometer vector magnitude | 0.02 (0.01, 0.02) |
| Sensor temperature | -0.37 (-0.71, -0.03) |
| Sensor humidity | 0.15 (0.01, 0.29) |
| Station-measured temperature | -1.19 (-2.39, 0.01) |
| Wave 2 (vs. wave 1) | 4.12 (0.95, 7.29) |
| Time of the day (vs. afternoon) |  |
| Evening | 0.96 (-0.76, 2.68) |
| Morning | -1.30 (-3.28, 0.68) |
| Day of the week (vs. weekday) |  |
| Weekend | -4.12 (-6.94, -1.30) |
| O_3_* wave | -2.01 (-3.63, -0.39) |
|  |  |
| Random intercept standard deviation | 9.28 |
| MobiliSense study, 55 participants, 1726 blood pressure measurements  ^1^All models included the following variables: short-term exposure to all the air pollutants (except PM2.5), accelerometer vector magnitude, physical activity status, temperature (sensor and station based), relative humidity (measured by sensor), proportion of time spent in different contexts (home, motorized vehicle, and non-motorized vehicles), wave indicator, time of the day, day of the week, residence area, age, sex, education, employment, monthly alcohol consumption, body mass index, and medication against hypertension. | |
|  |  |

Table 3 demonstrates the interaction of effects between O_3_ and wave in relation to systolic blood pressure modelled in a random intercept model including the 55 participants who were present in both waves. The model was adjusted for all the air pollutants, and time-varying and time-invariant covariates.

| Table 4: Interaction of effects between O_3_ and wave for systolic blood pressure, identified from a fixed effect model^1^ | |
| --- | --- |
| Variables | Systolic blood pressure |
| Air pollutants |  |
| BC (unit: µg/m^3^) | 0.98 (0.43, 1.53) |
| NO (unit: 10 ppb) | -0.13 (-0.38, 0.12) |
| NO_2_ (unit: 10 ppb) | 0.15 (-0.76, 1.06) |
| CO (unit: 100 ppb) | 0.03 (-0.01, 0.07) |
| O_3_ (unit: 10 ppb) | 2.59 (1.46, 3.73) |
| Age (years) | -1.20 (-2.63, 0.23) |
| Body mass index | -0.33 (-0.50, -0.16) |
| Monthly alcohol consumption (units) | -0.29 (-0.45, -0.12) |
| Proportion of time at domicile | -2.18 (-3.89, -0.47) |
| Proportion of time in motorized transport | 1.25 (-2.90, 5.39) |
| Proportion of time in non-motorized transport | -1.23 (-5.84, 3.38) |
| Accelerometer vector magnitude | 0.02 (0.01, 0.02) |
| Sensor temperature | -0.46 (-0.74, -0.17) |
| Sensor humidity | 0.11 (-0.00, 0.23) |
| Station-measured temperature | -1.42 (-2.42, -0.41) |
| Wave 2 (vs. wave 1) | 7.36 (3.59, 11.13) |
| Time of the day (vs. afternoon) |  |
| Evening | 1.87 (0.45, 3.29) |
| Morning | -1.34 (-3.00, 0.32) |
| Day of the week (vs. weekday) |  |
| Weekend | -3.70 (-5.93, -1.47) |
| O_3_* wave | -2.29 (-3.72, -0.85) |
| MobiliSense study, 55 participants, 1726 blood pressure measurements  ^1^All models included the following variables: short-term exposure to all the air pollutants (except PM2.5), accelerometer vector magnitude, physical activity status, temperature (sensor and station based), relative humidity (measured by sensor), proportion of time spent in different contexts (home, motorized vehicle, and non-motorized vehicles), wave indicator, time of the day, day of the week, age, monthly alcohol consumption, and body mass index. | |

Table 4 describes a fixed effect model that assessed the interaction of effects between O_3_ and wave in relation to systolic blood pressure. The model was estimated only among the participants who were present both in wave 1 and wave 2 of the MobiliSense study. The model was adjusted for air pollutants and time varying covariates.

| Table 5: Interaction of effects between black carbon and station-measured temperature in relation to systolic and diastolic blood pressure, identified from a fixed effect model^1^ | | |
| --- | --- | --- |
| Variables | Systolic blood pressure | Diastolic blood pressure |
| Air pollutants |  |  |
| BC (unit: µg/m^3^) | 0.69 (0.35, 1.04) | 0.21 (-0.09, 0.50) |
| NO (unit: 10 ppb) | -0.01 (-0.13, 0.11) | 0.02 (-0.08, 0.12) |
| NO_2_ (unit: 10 ppb) | 0.19 (-0.36, 0.74) | 0.42 (-0.04, 0.89) |
| CO (unit: 100 ppb) | 0.001 (-0.03, 0.03) | -0.04 (-0.06, -0.01) |
| O_3_ (unit: 10 ppb) | 1.42 (0.81, 2.03) | 0.47 (-0.04, 0.99) |
| Age (years) | -1.43 (-2.75, -0.10) | -1.63 (-2.74, -0.51) |
| Body mass index | -0.33 (-0.49, -0.18) | -0.22 (-0.35, -0.08) |
| Monthly alcohol consumption (units) | -0.31 (-0.47, -0.16) | -0.12 (-0.25, 0.01) |
| Proportion of time at domicile | -2.51 (-3.55, -1.47) | -2.43 (-3.31, -1.55) |
| Proportion of time in motorized transport | 0.08 (-2.30, 2.46) | -2.93 (-4.94, -0.92) |
| Proportion of time in non-motorized transport | -0.40 (-2.94, 2.14) | -0.02 (-2.16, 2.13) |
| Accelerometer vector magnitude | 0.02 (0.01, 0.02) | 0.002 (-0.00, 0.01) |
| Sensor temperature | -0.34 (-0.53, -0.15) | -0.13 (-0.28, 0.03) |
| Sensor humidity | 0.03 (-0.05, 0.11) | 0.01 (-0.05,0.08) |
| Station-measured temperature | -2.00 (-2.75, -1.25) | -1.14 (-1.77, -0.50) |
| Wave 2 (vs. wave 1) | 4.17 (1.40, 6.94) | 3.51 (1.18, 5.85) |
| Time of the day (vs. afternoon) |  |  |
| Evening | 1.54 (0.71, 2.37) | 0.91 (0.21, 1.61) |
| Morning | -0.74 (-1.76, 0.29) | 0.29 (-0.58, 1.15) |
| Day of the week (vs. weekday) |  |  |
| Weekend | -3.45 (-5.50, -1.40) | -2.33 (-4.06, -0.60) |
| BC * station-measured temperature | 0.64 (0.32, 0.96) | 0.34 (0.07, 0.61) |
| MobiliSense study, 235 participants, 4354 blood pressure measurements  ^1^All models included the following variables: short-term exposure to all the air pollutants (except PM2.5), accelerometer vector magnitude, physical activity status, temperature (sensor and station based), relative humidity (measured by sensor), proportion of time spent in different contexts (home, motorized vehicle, and non-motorized vehicles), wave indicator, time of the day, day of the week, age, monthly alcohol consumption, and body mass index. | | |

Table 5 describes the fixed model that assessed the interaction of effects between BC and station-measured temperature in relation to systolic and diastolic blood pressure. The model was adjusted for air pollutants and time varying covariates.

| Table 6: Interaction of effects between BC and station-measured temperature in relation to systolic and diastolic blood pressure, identified from the selected random coefficient model^1^ | | |
| --- | --- | --- |
| Variables | Systolic blood pressure | Diastolic blood pressure |
| Air pollutants |  |  |
| BC (unit: µg/m^3^) | 0.59 (0.23, 0.95) | 0.17 (-0.14, 0.48) |
| NO (unit: 10 ppb) | 0.14 (-0.08, 0.36) | -0.01 (-0.21, 0.19) |
| NO_2_ (unit: 10 ppb) | 0.69 (-0.26, 1.62) | 0.74 (-0.00, 1.48) |
| CO (unit: 100 ppb) | -0.01 (-0.04, 0.03) | -0.03 (-0.06, -0.01) |
| O_3_ (unit: 10 ppb) | 0.91 (-0.52, 2.35) | 0.38 (-0.50, 1.27) |
| Sex (vs. female) |  |  |
| Male | 8.28 (5.17, 11.38) | 8.63 (6.03, 11.24) |
| Age (years) | 0.46 (0.26, 0.67) | 0.31 (0.14, 0.48) |
| Body mass index | 0.04 (-0.14, 0.22) | 0.03 (-0.11, 0.18) |
| Physical activity (vs. no) |  |  |
| Yes | -0.53 (-3.14, 2.09) | -1.74 (-3.91, 0.43) |
| Monthly alcohol consumption (units) | -0.05 (-0.18, 0.07) | -0.002 (-0.11, 0.10) |
| Education (vs. higher than high school) |  |  |
| Equal to high school | -0.38 (-3.85, 3.08) | -0.45 (-3.37, 2.46) |
| Lower than high school | -0.86 (-7.61, 5.88) | -0.27 (-5.87, 5.33) |
| Residence (vs. far suburbs) |  |  |
| Close suburbs | 0.60 (-6.21, 7.42) | 0.88 (-4.90, 6.66) |
| Paris | -0.87 (-8.46, 6.73) | 1.48 (-4.94, 7.89) |
| Medication against  hypertension (vs. no hypertension) |  |  |
| Not medicated | 19.72 (10.53, 28.92) | 15.26 (7.57, 22.95) |
| Regularly medicated | 4.08 (-1.70, 9.86) | 2.40 (-2.50, 7.31) |
| Employment (vs. stable employment) |  |  |
| Retired | -4.06 (-8.61, 0.50) | -1.57 (-5.40, 2.26) |
| Unemployed | 8.02 (1.86, 14.18) | 3.85 (-1.19, 8.90) |
| Unstable employment | -0.15 (-4.74, 4.44) | 1.13 (-2.62, 4.89) |
| Other employment | -3.29 (-6.90, 0.32) | -2.29 (-5.29, 0.72) |
| Proportion of time at domicile | -2.54 (-3.70, -1.38) | -2.74 (-3.72, -1.76) |
| Proportion of time in motorized transport | -0.99 (-3.41, 1.42) | -2.59 (-4.69, -0.48) |
| Proportion of time in non-motorized transport | 0.52 (-2.07, 3.11) | 0.01 (-2.20, 2.23) |
| Accelerometer vector magnitude | 0.02 (0.02, 0.02) | 0.003 (0.00, 0.01) |
| Sensor temperature | -0.18 (-0.40, 0.04) | -0.06 (-0.24, 0.13) |
| Sensor humidity | 0.02 (-0.07, 0.10) | 0.03 (-0.04, 0.10) |
| Station-measured temperature | -1.47 (-2.32, -0.63) | -1.02 (-1.73, -0.31) |
| Wave 2 (vs. wave 1) | 0.66 (-0.86, 2.18) | 0.44 (-0.81, 1.68) |
| Time of the day (vs. afternoon) |  |  |
| Evening | 0.88 (-0.05, 1.81) | 0.71 (-0.08, 1.49) |
| Morning | -0.94 (-2.07, 0.19) | 0.34 (-0.62, 1.30) |
| Day of the week (vs. weekday) |  |  |
| Weekend | -2.97 (-5.08, -0.87) | -1.64 (-3.39, 0.11) |
| BC * station-measured temperature | 0.23 (-0.12, 0.58) | 0.37 (0.07, 0.67) |
|  |  |  |
| Random intercept standard deviation | 19.47 | 12.51 |
| Random slopes standard deviation |  |  |
| NO_2_ | 3.90 | 2.83 |
| NO | 0.62 | 0.72 |
| O_3_ | 7.68 | 3.66 |
| Residual standard deviation | 10.48 | 9.10 |
| MobiliSense study, 235 participants, 4354 blood pressure measurements  ^1^All models included the following variables: short-term exposure to all the air pollutants (except PM2.5), accelerometer vector magnitude, physical activity status, temperature (sensor and station based), relative humidity (measured by sensor), proportion of time spent in different contexts (home, motorized vehicle, and non-motorized vehicles), wave indicator, time of the day, day of the week, residence area, age, sex, education, employment, monthly alcohol consumption, body mass index, and medication against hypertension. | | |
|  |  |  |

Table 6 describes the random coefficient model that was used to assess the interaction of effect between BC and station-measured temperature in relation to systolic and diastolic blood pressure. The model was adjusted for air pollutants, and time-invariant and time-varying covariates.

| Table 7: Associations between air pollutants and systolic and diastolic blood pressure, estimated from separate single air pollutant random intercept models (air pollutants not adjusted for each other)^1^ | | |
| --- | --- | --- |
| Models tested | Systolic blood pressure | Diastolic blood pressure |
| Model 1: BC (unit: µg/m^3^) | 0.63 (0.30, 0.97) | 0.26 (-0.06, 0.49) |
| Model 2: NO (unit: 10 ppb) | 0.04 (-0.07, 0.14) | 0.03 (-0.06, 0.12) |
| Model 3: NO_2_ (unit: 10 ppb) | 0.16 (-0.34, 0.66) | 0.37 (-0.05, 0.78) |
| Model 4: CO (unit: 100 ppb) | 0.01 (-0.02, 0.04) | -0.03 (-0.05, -0.00) |
| Model 5: O_3_ (unit: 10 ppb) | 1.21 (0.06, 1.82) | 0.41 (-0.10, 0.91) |
| MobiliSense study, 235 participants, 4354 blood pressure measurements  ^1^All models included the following variables: accelerometer vector magnitude, physical activity status, temperature (sensor and station based), relative humidity (measured by sensor), proportion of time spent in different contexts (home, motorized vehicle, and non-motorized vehicles), wave indicator, time of the day, day of the week, residence area, age, sex, education, employment, monthly alcohol consumption, body mass index, and medication against hypertension. | | |

Table 7 describes ten different models that assessed the association between a single air pollutant and systolic blood pressure or diastolic blood pressure. All the models were adjusted for time-invariant and time-varying covariates.

| Table 8: Associations between BC and systolic and diastolic blood pressure, predicted from the models for the deciles of the z-score of station-measured temperatures^1^ | | |
| --- | --- | --- |
| Deciles | Systolic blood pressure | Diastolic blood pressure |
| Minimum | -0.49 (-1.27, 0.29) | -0.46 (-1.12, 0.19) |
| 1^st^ decile | 0.09 (-0.42, 0.60) | -0.13 (-0.56, 0.29) |
| 2^nd^ decile | 0.32 (-0.11, 0.74) | -0.00 (-0.35, 0.35) |
| 3^rd^ decile | 0.49 (0.11, 0.87) | 0.10 (-0.22, 0.42) |
| 4^th^ decile | 0.61 (0.25, 0.97) | 0.17 (-0.14, 0.47) |
| 5^th^ decile | 0.72 (0.37, 1.07) | 0.23 (-0.07, 0.53) |
| 6^th^ decile | 0.90 (0.53, 1.26) | 0.33 (0.02, 0.64) |
| 7^th^ decile | 1.07 (0.66, 1.47) | 0.43 (0.09, 0.77) |
| 8^th^ decile | 1.36 (0.85, 1.87) | 0.60 (0.17, 1.02) |
| 9^th^ decile | 1.76 (1.07, 2.46) | 0.83 (0.25, 1.41) |
| 10^th^ decile | 2.86 (1.58, 4.14) | 1.46 (0.39, 2.52) |
| MobiliSense study, 235 participants, 4354 blood pressure measurements  ^1^All models included the following variables: short-term exposure to all the air pollutants (except PM2.5), accelerometer vector magnitude, physical activity status, temperature (sensor and temperature from Meteo France), an interaction term between BC and station-measured temperature, relative humidity (measured by sensor), proportion of time spent in different contexts (home, motorized vehicle and non-motorized vehicles), wave indicator, time of the day, day of the week, residence area, age, sex, education, employment, monthly alcohol consumption, body mass index, and medication against hypertension | | |

Table 8 presents the estimates of association between BC and systolic and diastolic blood pressure predicted from the models for the deciles of the z-score of station-measured temperatures. These models were adjusted for time-invariant and time-varying covariates.
